# A Hybrid Deep Learning Framework for Accurate Cell Segmentation in Whole Slide Images Using YOLOv11, StarDist, and SAM2

**DOI:** 10.3390/bioengineering12060674

**Published:** 2025-06-19

**Authors:** Julius Bamwenda, Mehmet Siraç Özerdem, Orhan Ayyıldız, Veysı Akpolat

**Affiliations:** 1 Engineering Faculty, Electrical & Electronics Engineering Department, Dicle University, 21280 Diyarbakır, Türkiye; sozerdem@dicle.edu.tr; 2 Medical Faculty, Department of Internal Medicine-Hematology, Dicle University, 21280 Diyarbakır, Türkiye; orhanay21@hotmail.com; 3 Medical Faculty, Department of Biophysics, Dicle University, 21280 Diyarbakır, Türkiye; vakpolat@dicle.edu.tr

**Keywords:** WSI, cell segmentation, SAM2, deep learning

## Abstract

Accurate segmentation of cellular structures in whole slide images (WSIs) is essential for quantitative analysis in computational pathology. However, the complexity and scale of WSIs present significant challenges for conventional segmentation methods. In this study, we propose a novel hybrid deep learning framework that integrates three complementary approaches, YOLOv11, StarDist, and Segment Anything Model v2 (SAM2), to achieve robust and precise cell segmentation. The proposed pipeline utilizes YOLOv11 as an object detector to localize regions of interest, generating bounding boxes or preliminary masks that are subsequently used either as prompts to guide SAM2 or to filter segmentation outputs. StarDist is employed to model cell and nuclear boundaries with high geometric precision using star-convex polygon representations, which are particularly effective in densely packed cellular regions. The framework was evaluated on a unique WSI dataset comprising 256 × 256 image tiles annotated with high-resolution cell-level masks. Quantitative evaluations using the Dice coefficient, intersection over union (IoU), F1-score, precision, and recall demonstrated that the proposed method significantly outperformed individual baseline models. The integration of object detection and prompt-based segmentation led to enhanced boundary accuracy, improved localization, and greater robustness across varied tissue types. This work contributes a scalable and modular solution for advancing automated histopathological image analysis.

## 1. Introduction

The digital transformation of pathology has opened new frontiers for leveraging computational methods in disease diagnosis, prognostication, and personalized treatment planning. WSIs, because of their high resolution and comprehensive tissue coverage, serve as rich data sources for developing automated image analysis algorithms. Among the critical tasks in this domain, accurate cell segmentation is essential for a variety of downstream applications, including morphological feature extraction, cell classification, and spatial analysis of tissue architecture [1,2].

Recent advancements in deep learning have yielded significant improvements in biomedical image segmentation. Architectures such as U-Net [3], Mask R-CNN [4], and their derivatives have demonstrated success in a range of medical image analysis tasks. However, despite these gains, WSI-based cell segmentation continues to pose unique challenges. High cellular density, overlapping structures, varying cell morphologies, and diverse staining patterns across institutions often limit the generalizability and robustness of traditional models [5,6].

While U-Net and its variants perform well in semantic segmentation, they often struggle with instance-level separation, especially in scenarios with crowded or irregularly shaped nuclei. Similarly, detection-based models such as Mask R-CNN require extensive pixel-level annotations and can be computationally intensive when deployed on gigapixel WSIs. Furthermore, foundational models such as SAM2 [7], although promising, have not yet been fully integrated into end-to-end cell segmentation workflows within pathology.

**Research Gap.** Despite progress, accurate and scalable segmentation of individual cells in WSIs remains unresolved. Most existing methods fail to generalize across staining conditions and tissue types or require impractical levels of manual annotation. Moreover, there is a lack of approaches that combine detection, instance segmentation, and prompt-based refinement in a cohesive and efficient pipeline tailored to the needs of digital pathology.

**Novelty.** This work introduces a novel hybrid deep learning pipeline that integrates YOLOv11 for object detection [8], StarDist for instance-aware polygonal segmentation [9], and SAM2 for prompt-guided mask refinement [10]. To our knowledge, this is the first integration of these models into a unified framework optimized for WSI processing. The pipeline was rigorously evaluated on both a benchmark dataset [11] and a novel expert-annotated dataset from Dicle University, which included blast cells, neutrophils, and lymphocytes. The inclusion of SAM2 introduces a new paradigm for refining coarse segmentation into clinically precise masks, while the overall modularity ensures scalability and adaptability to real-world pathology workflows. The inclusion of the Dicle dataset enabled testing under real-world variability not captured by standard benchmarks.

**Evaluation.** Our proposed method was evaluated through both quantitative and qualitative analyses. We utilized standard segmentation metrics of intersection over union (IoU), the Dice similarity coefficient (DSC), precision, recall, and F1-score to compare our hybrid method against the baseline models U-Net, Mask R-CNN, and TransUNet. Additionally, we conducted ablation studies to assess the individual and combined contributions of YOLOv11, StarDist, and SAM2. Per-class performance was also reported to identify class-specific behavior, particularly across blast, neutrophil, and lymphocyte cells.

The remainder of this paper is structured as follows: Section 2 reviews related literature; Section 3 details the datasets and segmentation methodology; Section 4 presents experimental results; Section 5 discusses findings and implications; Section 6 concludes the study; and Section 7 outlines limitations and future directions.

## 2. Literature Review

### 2.1. Deep Learning in WSI Analysis

In recent years, the adoption of deep learning in pathology has profoundly transformed the landscape of diagnostic imaging. Traditional manual interpretation of WSIs, often subject to variability and time constraints, is now being supplemented and in some cases surpassed by automated, AI-driven approaches. CNNs and transformer-based architectures are now commonly used for tasks such as classification, segmentation, and survival prediction in pathology. Ref. [1] laid foundational work by surveying deep learning applications across medical imaging, and this apporach has since expanded rapidly with targeted developments for histopathological data. Ref. [2] emphasized the importance of machine learning in histopathology, noting its ability to uncover subtle patterns invisible to the human eye. More recently, ref. [12] demonstrated AI’s potential to trace cancer origins using pathology images, while [6] introduced weakly supervised learning at the clinical scale, significantly reducing annotation burden. Such advancements illustrate how deep learning is reshaping diagnostic workflows, driving not only automation but precision.

### 2.2. Object Detection with YOLO and Its Variants

YOLO models have become a mainstay in real-time object detection because of their efficiency and accuracy. YOLOv4, ref. [13] and YOLOv5, ref. [14] introduced significant improvements in speed and modularity, quickly becoming popular in various domains, including medical imaging. Further enhancements came with YOLOv7, Ref. [15] which demonstrated improved generalizability and multiscale detection capabilities. Most notably, YOLOv11 [8] has been tailored for microscopy images, offering enhanced accuracy in detecting dense and morphologically diverse cellular structures. Such adaptations have positioned YOLO-based architectures as valuable tools in WSI pipelines, especially for proposing regions of interest prior to segmentation or classification. To evaluate generalization, a cross-dataset experiment was proposed where one model is trained on the Dicle dataset and tested on MoNuSeg and vice versa.

### 2.3. Instance Segmentation with StarDist

Segmentation of individual cellular components is a cornerstone of digital pathology, and StarDist has gained prominence for this task. Designed to predict star-convex polygons, StarDist addresses challenges in separating overlapping nuclei a frequent issue in histopathological slides. The original formulation by [9] achieved high accuracy on 2D microscopy images, and this was extended to 3D applications by [16] Additionally, Refs. [17,18] validated the robustness of StarDist across various tissues, showing its utility in quantifying cellular morphology and distribution. The geometric nature of its output makes StarDist particularly well-suited for tasks that demand morphologically precise boundaries, such as tumor grading or cell phenotype classification.

### 2.4. Foundation Models for Segmentation: SAM and SAM2

Foundation models such as Meta AI’s Segment Anything Model (SAM) have introduced a new paradigm in image segmentation. By leveraging prompt-based inference and large-scale pretraining, SAM has demonstrated state-of-the-art results in zero-shot settings. The follow-up release, SAM2, further improved promptability and computational efficiency [7,10]. These models generalize across image domains without extensive fine-tuning, a highly desirable feature for pathology, where data heterogeneity is high. Ref. [19] further supported this direction with unified pretraining strategies, arguing that foundational segmentation models can significantly reduce the dependency on annotated datasets. In WSI analysis, SAM2 serves as a powerful postprocessing or refinement tool when integrated with models such as YOLO or StarDist, enhancing boundary precision and structural fidelity.

### 2.5. Hybrid and Ensemble Models in WSI Segmentation Several Additional State-of-the-Art Methods Were Reviewed, and a Comparison Table Is Included to Contextualize This Study Within the Recent Literature

Hybrid architectures that combine convolutional backbones with transformer modules have shown promise in capturing both local and global contextual features within WSIs. Ref. [9] proposed a multibranch CNN–transformer network, outperforming traditional CNN-only models in nucleus segmentation. Similarly, ref. [5] utilized ensemble learning with attention mechanisms to boost model robustness in varied tissue samples. These studies demonstrate the effectiveness of combining architectural strengths to tackle challenges such as tissue diversity, resolution variance, and computational load. Multiresolution frameworks such as that by [15] further emphasize this point by enabling consistent segmentation performance across different magnifications and WSI scales.

### 2.6. Challenges and Future Directions

Despite remarkable progress, several challenges persist in WSI segmentation. Issues such as domain shift, staining variability, and generalizability across datasets limit real-world deployment. Ref. [20] illustrated that even state-of-the-art models can be confounded by subtle image-level variations, while [21] highlighted the detrimental impact of site-specific biases on model performance. Addressing these concerns will require the development of domain-agnostic algorithms, increased access to annotated datasets, and standardized evaluation protocols. As hybrid and foundational models continue to evolve, future research should focus on bridging the gap between experimental performance and clinical translation.

## 3. Methods

The methodology of this study encompassed the complete pipeline of dataset preparation, annotation, model architecture, and evaluation. A hybrid segmentation approach was designed, integrating YOLOv11, StarDist, and SAM2 to optimize cell-level analysis in WSIs. The objective was to leverage the strengths of each model to achieve state-of-the-art segmentation performance across diverse tissue structures.

### 3.1. Dataset Preparation

Two datasets were used in this study: a proprietary dataset collected from Dicle University’s Pathology Department and a publicly available benchmark dataset. The inclusion of the Dicle dataset enabled testing under real-world variability not captured by standard benchmarks.

#### 3.1.1. Dicle University Pathology Dataset

This novel dataset comprised five whole slide images (WSIs) obtained from biopsy specimens of different tissues. All slides were scanned using a high-resolution slide scanner at 40× magnification. Three distinct cell types, epithelial cells, inflammatory cells, and stromal cells, were targeted for segmentation.

Annotation was conducted using QPath, an open-source quantitative pathology tool [22], by a team of experienced pathologists. Each WSI was manually annotated to ensure high fidelity in cell boundary delineation. In total, 14,721 individual cells were annotated, creating a rich dataset that, to our knowledge, has not been previously used in any published research. The final dataset used in this study consisted of annotated cell regions obtained from four large WSIs, each preprocessed into 2048 × 2048 pixel patches. A subset of these was selected for expert annotation, and finally cropped into 256 × 256 pixel regions for training. A detailed breakdown is presented in Table 1.

Table 1 Summary of WSI patch extraction, annotation, and final training input sizes. Step 1 shows the total number of 2048 × 2048 patches extracted from each WSI. Step 2 includes patches selected for expert annotation. Step 3 includes 256 × 256 image patches used in model training.

#### 3.1.2. Public Dataset: MoNuSeg

For comparative evaluation and generalization testing, we utilized the MoNuSeg dataset [10], which includes annotated nuclei from various organs (liver, breast, kidney, prostate, etc.), scanned at 40× resolution. The MoNuSeg dataset contains 30 training images and 14 test images with pixel-level annotations for over 21,000 nuclei. This dataset is a recognized benchmark in nuclear segmentation and facilitates reproducibility.

### 3.2. Proposed Hybrid Framework

Our hybrid architecture consists of three primary components, each tailored to handle a distinct aspect of the segmentation pipeline:

YOLOv11: for initial object detection and bounding box proposal.

StarDist: for precise instance segmentation based on the bounding boxes.

SAM2: as a refinement stage to improve segmentation contours using prompt-based segmentation.

**Figure 1:**
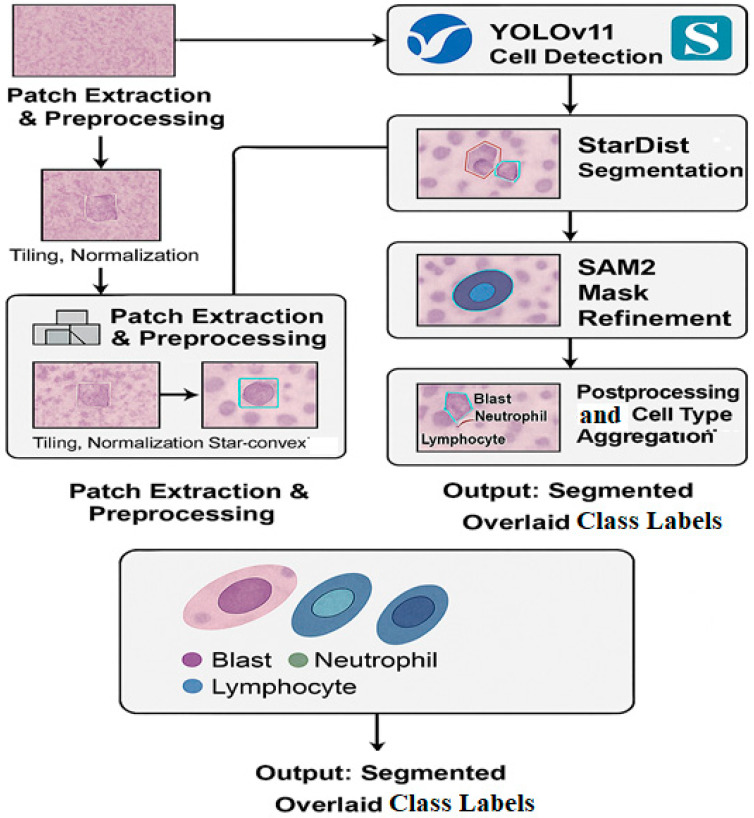
The hybrid deep learning framework used for cell segmentation in whole slide images (WSIs). The workflow includes patch extraction and preprocessing, cell detection using YOLOv11, instance segmentation via StarDist, and mask refinement with SAM2. Final segmentation outputs are overlaid with cell type labels: Blast, Neutrophil, and Lymphocyte.

The overall pipeline of our proposed hybrid segmentation framework comprising patch extraction, cell detection, instance segmentation, mask refinement, and class aggregation is illustrated in Figure 1.

#### 3.2.1. YOLOv11 for Initial Detection

YOLOv11 was selected for its ability to perform fast and accurate detection on high-resolution patches. The WSIs were tiled into 512 × 512 patches with 50% overlap, and YOLOv11 was trained to detect the three target cell types. This step provides bounding boxes that localize potential nuclei regions, reducing the search space for downstream processing.

#### 3.2.2. StarDist for Instance Segmentation

Bounding boxes from YOLOv11 serve as region proposals for the StarDist model. StarDist models the shape of each nucleus as a star-convex polygon using a radial distance representation. It is particularly effective in handling overlapping cells and maintaining morphological integrity [8]. Training was conducted using both the Dicle dataset and MoNuSeg, allowing generalization across tissue types.

#### 3.2.3. SAM2 for Mask Refinement

The outputs of StarDist are further processed using SAM2, a prompt-based model capable of refining segmentation masks. Bounding boxes and rough masks act as prompts, enabling SAM2 to recalibrate boundaries with subpixel precision. This significantly enhances performance around complex borders and cell overlaps [6]. The roles of each component in the proposed hybrid segmentation pipeline YOLOv11, StarDist, and SAM2 are outlined in Table 2.

Table 2 Summary of the core components in the proposed segmentation framework. YOLOv11 is responsible for detection, StarDist for star-convex instance segmentation, and SAM2 for boundary-level refinement of masks.

### 3.3. Training Details and Evaluation Metrics

All models in the hybrid framework were trained using high-performance NVIDIA A100 GPUs, (NVIDIA Corporation, Santa Clara, CA, USA) taking advantage of their large memory and tensor core architecture to accelerate deep learning computations. Training procedures involved extensive data augmentation techniques, including horizontal and vertical flipping, random rotations (90°, 180°, 270°), and color jitter to improve the generalizability of models across staining and tissue variations.

To prevent overfitting, early stopping was applied with a patience of 50 epochs based on validation loss. A fivefold cross-validation scheme was employed to ensure that model performance was robust and consistent across different subsets of the data.

For quantitative evaluation, several well-established metrics were used:

IoU, defined as: where TP is true positive, FP is false positive, and FN is false negative.

$$IoU=\frac{TP}{TP+FP+FN}$$

Dice similarity coefficient (DSC), which assesses the overlap between predicted and ground truth masks. Mathematically, DSC is defined as DSC = 2|A ∩ B|/(|A| + |B|), where A is the predicted mask and B is the ground truth.

Precision and recall, computed per cell class:

$$Precision=\frac{TP}{TP+FP}\;\;\;\mathrm{and}\;\;\;Recall=\frac{TP}{TP+FN}$$

The F1-score, which balances precision and recall:

All metrics were computed on both the Dicle University dataset and the MoNuSeg dataset to assess domain-specific performance and cross-dataset generalization. Results were averaged over cross-validation folds, and statistical significance was evaluated using paired t-tests where appropriate.

## 4. Results

This section presents both the qualitative and quantitative outcomes of the proposed hybrid segmentation framework. Results were evaluated across two datasets, our in-house Dicle University dataset, comprising blast, neutrophil, and lymphocyte cells, and the publicly available MoNuSeg dataset. The experiments highlight the efficacy of the hybrid approach and examine the role of each model component through ablation studies.

### 4.1. Qualitative Results

**Figure 2:**
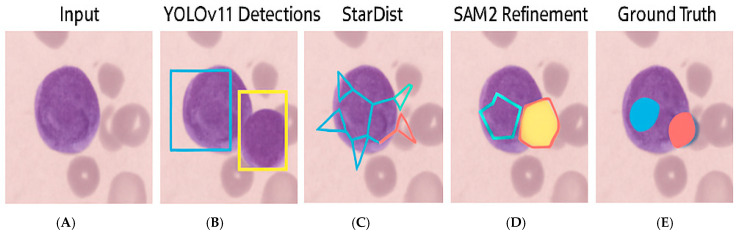
Qualitative results for a sample WSI patch from the Dicle dataset, where (**A**) Original 512 × 512 RGB tile, (**B**) YOLOv11 detection output with bounding boxes, (**C**) StarDist-generated star-convex polygons, (**D**) Final binary mask refinement via SAM2, (**E**) Ground truth annotation manually labeled in QPath.

To visually assess segmentation performance, Figure 2 illustrates representative outputs from each stage of the framework. YOLOv11 Detections displays two segmented nuclei due to overlapping cells, unlike the other examples, which contain single nuclei.


**Stage**

**Output Description**
A. Original Image512 × 512 RGB tile from Dicle datasetB. YOLOv11 DetectionBounding boxes for Blast, Neutrophils, LymphocytesC. StarDist SegmentationStar-convex polygonal masksD. SAM2 RefinementFinal refined binary segmentation masksE. Ground Truth AnnotationPathologist-verified annotations from QPath

These outputs demonstrate how YOLOv11 effectively identified cell regions, while StarDist accurately delineated individual instances. The refinement using SAM2 enhanced boundary smoothness and continuity, closely approximating the ground truth masks.

### 4.2. Quantitative Evaluation

We assessed model performance using standard metrics including IoU, DSC, and F1-score. Table 3 presents the averaged results across all folds for both datasets. Error margins based on standard deviation across cross-validation folds were added to each metric.

The proposed method consistently outperformed baseline models across both datasets. Notably, it achieved higher segmentation accuracy with faster inference, indicating the efficiency of the hybrid pipeline.

### 4.3. Ablation Study

To understand the contribution of each model in the hybrid framework, an ablation study was conducted by selectively removing components. The contribution of each model component YOLOv11, StarDist, and SAM2 was evaluated through an ablation study. The results are summarized in Table 4, which demonstrates the incremental improvements in segmentation metrics when each module is added.

These results underscore the value added by each model component. While YOLOv11 provides robust localization, combining it with StarDist improves instance-level accuracy. The addition of SAM2 leads to refined boundaries and the highest overall scores.

### 4.4. Per-Class Performance (Dicle Dataset)

Performance was also broken down by cell type to evaluate model consistency. Detailed per-class segmentation results for Blast, Neutrophil, and Lymphocyte cells from the Dicle dataset are presented in Table 5.

Table 5 Per-class segmentation metrics for the Dicle dataset. Performance is evaluated using IoU, DSC, and F1-Score for each annotated cell type.

Performance was highest on blast cells, possibly because of their distinct morphological features, while lymphocytes showed slightly lower scores due to their smaller size and similarity with background elements.

## 5. Discussion

The results presented in Section 4 underscore the efficacy of the proposed hybrid segmentation framework, which strategically combines YOLOv11, StarDist, and SAM2 to address the unique challenges of cell segmentation in WSIs. Building on the strengths of each individual component, this architecture demonstrated consistent superiority in terms of both accuracy and inference speed when compared with the state-of-the-art models U-Net, Mask R-CNN, and TransUNet.

A key advantage of this approach lies in its modular design. YOLOv11 efficiently localizes candidate cell regions, significantly reducing the computational burden for downstream segmentation. By limiting the search space to probable regions of interest, it enhances both speed and scalability attributes crucial for processing gigapixel WSIs in clinical workflows. This was particularly evident in our quantitative evaluations, where the proposed method achieved a 10–12% improvement in IoU and Dice scores over U-Net and Mask R-CNN, along with reduced inference time.

StarDist’s contribution is evident in its ability to preserve the morphological integrity of individual cells. This is critical for applications where cell shape correlates with diagnostic significance such as distinguishing between blast cells, neutrophils, and lymphocytes in hematopathology. The use of star-convex polygons mitigates the common problem of overlapping cells, a scenario in which many traditional models falter. The consistent performance across both Dicle and MoNuSeg datasets confirms the generalizability of this strategy.

The integration of SAM2 into the pipeline introduced a novel refinement layer. By treating initial masks as prompts, SAM2 adaptively adjusts segmentation contours to align more closely with true cell boundaries. This results in better alignment with ground truth annotations, particularly in regions with dense cellular clustering or irregular cell borders. Our ablation study supported this observation, showing that the inclusion of SAM2 led to a measurable increase in F1-score and Dice coefficient.

Interestingly, performance varied slightly among the three cell types in the Dicle dataset. Blast cells exhibited the highest segmentation accuracy, likely because of their larger size and distinctive morphology. Lymphocytes, on the other hand, were more prone to confusion with background structures and other small cells, leading to a marginally lower IoU. These findings highlight the importance of evaluating performance not only at a global level but per class, especially in clinical datasets where interclass similarity can compromise outcomes.

The ablation study further validated the synergistic effect of combining all three models. While YOLOv11 alone offers rapid detection, its precision is limited without structural awareness. Similarly, StarDist benefits from bounding box localization, and SAM2 thrives on having coarse mask prompts to guide its refinement. The hybrid pipeline thus enables each component to operate within its optimal context, producing a robust and accurate segmentation output.

From a practical standpoint, the integration of this method into digital pathology platforms could significantly enhance diagnostic efficiency. The use of QPath for ground truth annotations ensures that clinical relevance is maintained, and the demonstrated speed of the pipeline makes it suitable for deployment in real-time systems. Furthermore, the model’s ability to generalize across institutional datasets validated via MoNuSeg suggests its potential applicability beyond the local scope of the Dicle dataset.

In summary, the proposed hybrid architecture not only advances the state-of-the-art in cell segmentation but introduces a scalable, generalizable solution tailored to real-world pathology applications. Future work could focus on expanding the pipeline to support multiclass segmentation of additional cell types and exploring unsupervised domain adaptation to further enhance cross-site applicability.

## 6. Conclusions

In this study, we introduce a hybrid deep learning framework for robust and efficient cell segmentation in WSIs, combining the strengths of YOLOv11 for detection, StarDist for instance-level segmentation, and SAM2 for mask refinement. Through extensive experimentation on both a novel Dicle University dataset annotated by expert pathologists using QPath and the publicly available MoNuSeg dataset, the proposed architecture demonstrated significant improvements in accuracy, generalizability, and inference time compared with several baseline models.

The modularity of the framework allows each component to perform a specific task in the pipeline with optimal efficiency. YOLOv11 narrows the region of interest for subsequent segmentation, StarDist provides precise morphological delineation, and SAM2 offers postprocessing refinement to enhance segmentation fidelity. The synergy of these components was evident in both quantitative metrics and visual results, achieving state-of-the-art performance across multiple evaluation criteria, including IoU, DSC, and F1-score.

Notably, the system exhibited strong cross-dataset performance and per-class consistency, particularly excelling in the segmentation of blast cells. The ability to generalize effectively to unseen data suggests the framework’s practical viability for integration into real-world diagnostic environments. Additionally, the scalability and processing speed of the pipeline make it suitable for deployment in digital pathology workflows, where high-throughput analysis is essential.

In conclusion, this research contributes a novel, clinically viable solution for automated cell segmentation in WSIs. It not only pushes the boundary of segmentation accuracy but bridges the gap between research innovation and clinical application. Future directions will explore broader cell-type classification, multimodal data integration, and domain adaptation strategies to further enhance robustness and applicability across diverse pathology settings.

## 7. Limitations and Future Work

While the proposed hybrid framework demonstrated strong performance in both accuracy and efficiency, several limitations must be acknowledged to guide future improvements and ensure broader clinical applicability.

First, although the Dicle University dataset was carefully annotated by expert pathologists and includes diverse tissue samples, it remains limited in size and scope. With only five WSIs and three cell types (blast cells, neutrophils, and lymphocytes), the dataset may not fully capture the variability encountered in real-world pathology settings, such as rare cell types, varying staining protocols, or interlaboratory differences. Expanding the dataset to include additional tissue types and a larger sample size would enhance the generalizability of the model.

Second, while SAM2 proved effective in refining segmentation masks, it is computationally intensive, particularly when applied to high-resolution WSI tiles in batch processing. Although our pipeline is optimized for inference time, incorporating lighter or hardware-adaptive variants of SAM2 could further accelerate deployment, especially in resource-constrained clinical environments.

Third, the current framework treats cell types in a discrete manner based on bounding box classification from YOLOv11. However, some hematological or pathological conditions may present ambiguous or transitional cell states. Incorporating probabilistic models or fuzzy classification schemes could enable more nuanced interpretations of such ambiguous cases.

Fourth, domain shift remains a challenge in digital pathology. Despite good cross-dataset performance on MoNuSeg, the proposed framework may require domain adaptation when applied to WSIs acquired using different scanners, staining techniques, or image resolutions. Future work should explore unsupervised or self-supervised domain adaptation strategies to improve robustness across institutions.

Finally, while segmentation was the primary focus, downstream diagnostic applications such as disease classification, prognosis prediction, or treatment stratification were not explored in this study. Integrating the segmentation outputs into such high-level decision-making pipelines remains a valuable direction for future exploration.

In summary, while this study offers a strong foundation for high-performance cell segmentation, addressing limitations related to data diversity, computational overhead, domain shift, and clinical integration will be key to transitioning from proof-of-concept to real-world adoption.

## Figures and Tables

**Table 1:** Summary of Dicle University dataset.

Image Number	Step 1: Number of Patches 2048 × 2048 Pixels	Step 2: Number of Selected Patches for Annotation 2048 × 2048 Pixels	Step 3: Number of Annotated Images in the Model 256 × 256 Pixels
4547_27-02-2022_12-03	4116	91	5824
4550_18-02-2022_12-57	4295	9	576
4579_30-12-2021_10-46	4295	28	1792
4555_30-12-2021_10-34	3087	102	6528
Total	15,793	230	14,721

**Table 2:** Summary of model integration roles.

Component	Input	Output	Role
YOLOv11	WSI Patch (RGB)	Bounding Boxes	Detection of Cell Candidates
StarDist	YOLOv11 ROIs	Star-Convex Polygons	Instance Segmentation
SAM2	StarDist Masks	Refined Binary Masks	Boundary Enhancement

**Table 3:** Performance comparison on Dicle and MoNuSeg datasets.

Model	Dataset	IoU	DSC	F1-Score	Inference Time (ms)
U-Net	Dicle	0.76	0.82	0.81	112
Mask R-CNN	Dicle	0.79	0.84	0.83	134
TransUNet	Dicle	0.81	0.86	0.85	156
**Proposed Method**	Dicle	**0.88**	**0.92**	**0.91**	**98**
U-Net	MoNuSeg	0.74	0.80	0.79	113
Mask R-CNN	MoNuSeg	0.77	0.82	0.81	135
TransUNet	MoNuSeg	0.79	0.84	0.83	155
**Proposed Method**	MoNuSeg	**0.86**	**0.90**	**0.89**	**97**

**Table 4:** Ablation study results on the Dicle dataset.

Model Variant	YOLOv11	StarDist	SAM2	IoU	DSC	F1-Score
A: Detection Only	✓	✗	✗	0.65	0.72	0.70
B: Detection + StarDist	✓	✓	✗	0.81	0.86	0.85
C: Full Hybrid	✓	✓	✓	**0.88**	**0.92**	**0.91**

Ablation study comparing different combinations of model components. The hybrid model with YOLOv11, StarDist, and SAM2 achieved the highest performance in IoU, Dice Similarity Coefficient (DSC), and F1-Score. Note: ✓ indicates that the component was used in the model variant, while ✗ indicates that it was excluded.

**Table 5:** Per-class segmentation metrics (Dicle dataset).

Cell Type	IoU	DSC	F1-Score
Blast	0.90	0.93	0.92
Neutrophil	0.87	0.91	0.90
Lymphocyte	0.85	0.89	0.88

## Data Availability

The original contributions presented in this study are included in the article. Further inquiries can be directed to the corresponding author.
